# Comparison of vaccine efficacy must be based on good clinical data–Authors’ reply

**DOI:** 10.1016/j.lanwpc.2022.100413

**Published:** 2022-02-27

**Authors:** Nur Asheila Abdul Taib, Dhesi Baha Raja, Alvin Kuo Jing Teo, Adeeba Kamarulzaman, Timothy William, Arvinder-Singh HS, Siti Aisah Mokhtar, Choo-Yee Ting, Wei Aun Yap, Michelle Yoon Kim Chan, Lidwina Edwin Amir

**Affiliations:** a AINQA Health Sdn. Bhd., Lot 7.01 B & C, Menara BRDB,285 Jalan Maarof, Bukit Bandaraya, Kuala Lumpur 59000, Malaysia; b Saw Swee Hock School of Public Health, National University of Singapore, National University Health System, Singapore; c Department of Medicine, Faculty of Medicine, University of Malaya, Kuala Lumpur, Malaysia; d Gleneagles Hospital, Kota Kinabalu, Sabah, Malaysia; e Infectious Diseases Society Kota Kinabalu Sabah-Menzies School of Research Clinical Research Unit, Kota Kinabalu, Sabah, Malaysia; f Queen Elizabeth Hospital-Clinical Research Centre, Ministry of Health, Kota Kinabalu, Malaysia; g Department of Community Health, Faculty of Medicine, Universiti Kebangsaan Malaysia, Malaysia; h Faculty of Medicine and Health Sciences, Universiti Putra Malaysia, Malaysia; i Faculty of Computing and Informatics, Multimedia University, Malaysia; j Quanticlear Solutions Sdn. Bhd., Malaysia

We thank the author of the letter for the interest in our article.^1^ The author commented that the recipients of the three vaccine types might not be comparable in the national COVID-19 mortality line list. The author suggested that this limitation could be addressed by applying statistical tests to the baseline characteristics of the three groups, especially the number and severity of co-morbidities. As the dataset involved only those with the outcome of interest (COVID-19 deaths), we could not meaningfully compare the baseline characteristics of all vaccine recipients in the country. We were also limited by the official released data and information on comorbidities (available in binary form (yes/no)), in which we have alluded in the paper that we could not further describe the risk of deaths by the types of comorbidities. Granular information on the severity of other comorbidities, especially in the presence of >1 conditions, may be complex, requiring a larger sample size to meaningfully group people with similar comorbidities, challenging to ascertain accurately, and affected by temporal bias (sequence and interplay of severe COVID-19 and its relationship with the severity of other comorbidities). Therefore, given the limitations, we did not further compare the performance of the different vaccine types in preventing COVID-19 death statistically but only described death rates by vaccine types and status between February and September 2021. Future research is recommended to determine statistically meaningful evidence comparing the effectiveness of vaccines and other predictors of breakthrough infections and outcomes.

We agree with the author that the comparison of vaccine efficacy must be based on good clinical data. As vaccine efficacy could only be established in a controlled environment such as a clinical trial, we could not do so in our study. We also acknowledged in the paper the lack of detailed information regarding vaccine recipients who remained healthy and/or those who did not succumb to the disease, thereby limiting our ability to infer vaccine effectiveness in the Malaysian populations. However, inferences on vaccine effectiveness in Malaysia were reported by Suah et al. using datasets with relatively more data points concurred with the findings in our study.^2^ The data we analysed encompassed all COVID-19 deaths that occurred and were recorded in Malaysia during the analysis period (the line list is still being updated daily). While it lacks granularity in some respects (e.g., types and severity of comorbidities, socioeconomic factors), we opined that the data is the closest representation possible of the actual scenario in the country and holds crucial information needed to understand and respond to the pandemic.

The COVID-19 vaccination programme in Malaysia was implemented in phase 1 (frontline workers), phase 2 (older persons, disabled, and high-risk individuals), and phase 3 (other adults aged 18 and above). BNT162b2 was the first vaccine to be initiated in Malaysia and was the predominant vaccine used for phase 1 and the early half of phase 2 between February 2021 and May 2021.^2^^,^^3^ As described in the paper, the subsequent allocation depended on vaccine availability. The inactivated vaccine was mainly used in June and July 2021 before the BNT162b2 supply resumed from August 2021 onwards.^3^ The author has rightly pointed out that there is good evidence now that immunity against COVID-19, both infection-acquired (especially neutralising antibodies) and vaccine-acquired, wanes over time.^4^, ^5^, ^6^ Considering that BNT162b2 was initiated earlier in Malaysia, waning vaccine effectiveness would have impacted the mRNA-based vaccine more than the inactivated vaccine.

In our dataset, the median time from being fully vaccinated (2 weeks after the second dose) to death were 10 weeks for the recipients of BNT162b2 and 11 weeks for the recipients of the inactivated vaccine. While we did not analyse immunological data, the higher mortality rates among recipients of inactivated vaccines also coincided with the World Health Organization's recommendation to prioritise an additional dose of COVID-19 vaccine for the recipients of inactivated vaccines aged above 60 at the time of data analysis and reporting. Despite the reduction in vaccine effectiveness over time and their protection against emerging variants of SARS-CoV-2, encouraging reports on the protective effects of a 3-doses homologous/heterologous booster regimen and among convalescents who were fully vaccinated further support the booster practice adopted by many countries worldwide.^7^, ^8^, ^9^ Nevertheless, vaccine inequity remains a conundrum that will likely undermine global health and economic recoveries. Thus, global solidarity in sharing information and resources, especially vaccines, cannot be further emphasised.

We observed higher mortality rates among those fully vaccinated with inactivated vaccines than those who were partially vaccinated after accounting for age between 25 August and 14 September 2021. While the observations were atypical, the difference in the age-standardised mortality rates (ASMR) between the 2 groups was not statistically significant. As we further stratified the analysis by the presence of comorbidities, higher ASMR was observed among those with comorbidities in the partially and fully vaccinated groups, respectively.^1^ However, we acknowledge that there may be residual confounding that we could not account for. Based on the dataset analysed in this study, we could not postulate anecdotal evidence to explain the unusual observations reported during the period of interest.

Finally, the author concluded that in actual community use, the total COVID-19 deaths per million was lower in Lao People's Democratic Republic (hereinafter Laos), which mainly administered inactivated vaccines compared to Singapore that rolled out predominantly mRNA vaccines. Nonetheless, cross-country comparisons of vital COVID-19 indicators are complex due to the multifarious factors that must be considered. Using case fatality rate (CFR) as a case in point, COVID-19’s CFR in Laos when 49% of the total population was fully vaccinated, was approximately 0.4%. When 49% of the total population in Singapore was fully vaccinated, COVID-19 CFR in Singapore was 0.06%.^10^^,^^11^ Such comparisons are, however, marred by huge complexities such as the different transmission rates, the severity of the outbreak, the circulating variants, the restrictions imposed and public adherence to it, health system and reporting capacities, and the impact of other non-pharmaceutical interventions which could lead to the interference of ecological fallacy. Nonetheless, data on COVID-19 deaths attributable to mostly unvaccinated/not fully vaccinated people were consistently reported by Laos, Singapore, and beyond.^12^, ^13^, ^14^

## Declaration of interests

None.
